# Supplementary material for: Optimization control of all-terrain rescue lift vehicle safety performance based on state feedback
Source: PLoS One. 2025 May 9;20(5):e0323293. doi: 10.1371/journal.pone.0323293 (PMC12064021; doi:10.1371/journal.pone.0323293)
Supplement: Attachment — Submitted filename: Point-by-point response.pdf [file pone.0323293.s002.pdf]

Dear PhD. Zhang,

Thanks for your letter and for reviewers' comments concerning our manuscript entitled "Optimization control of all-terrain rescue lift vehicle safety performance based on state feedback" (Manuscript Number: PONE-D-24-59867). Those comments are all valuable and helpful for revising and improving our paper. We have studied all comments carefully and have made conscientious correction. During the revision process, we have also made adjustments to the manuscript format and related documents to ensure compliance with PLOS ONE's style requirements. Additionally, regarding the discrepancy in the funding information provided in the "Funding Information" and "Financial Disclosure" sections that you pointed out, we have corrected the relevant details in our submission. The main corrections in the paper and the responds to the reviewers' comments are as flowing.

Reviewer Comments & Author Responses

Reviewer Reports on the Initial Version:

Reviewer # 1

1. The images in the text did not display properly. Please ensure that there are no formatting errors in the final submitted version.
2. Table 1 should have 3 columns, each column consisting of parameter name, parameter meaning, and parameter value.
3. In the "Steering control system optimization design", a Linear 2-degree-of-freedom model was adopted, which means that this article only considers sideslip and yaw at the theoretical level, without considering roll and pitch. But the rollover caused by vehicle roll is also a serious problem. Is the dynamic model used in this article reasonable?
4. It is recommended to conduct tests under classic serpentine and hook conditions to verify the effectiveness of the proposed method in this paper.
5. For experimental results, in addition to visualized curves, it is recommended to provide quantifiable indicator results.
6. Why is PID used as the optimization controller in the article, and what are the advantages of this method compared to other methods?
7. This article uses existing dynamic models and control methods, and the innovation of this article is not clear enough.
8. The Introduction section lacks a comprehensive summary of existing research. It is recommended to cite "A review on reinforcement learning based highway autonomous vehicle control" and "Longitudinal and lateral control methods from single vehicle to autonomous platform".

Reviewer # 2

The manuscript investigates the steering stability of the XZJ5240JQZ30 all-terrain rescue lift vehicle under Double Line Change conditions. It develops a state-feedback PID optimization control system based on a two-degree-of-freedom vehicle model to enhance the vehicle's stability during steering maneuvers. Simulation results demonstrate that the proposed control system effectively improves the vehicle's stability and steering performance at both high and low speeds.

Below are my comments and suggestions.

1. Methodological aspects

1) Model Assumptions: The linear two-degree-of-freedom (2DOF) model is used for vehicle stability analysis. While this model is a good starting point, it may not fully capture the complex dynamics of a heavy rescue vehicle, especially under extreme conditions. Suggestion: Consider incorporating a more sophisticated model, such as a nonlinear model or a 3DOF model, to better represent the vehicle's behavior under various steering and speed scenarios.

2) Parameter Identification: The paper does not detail how the parameters for the mathematical models were identified or validated. Suggestion: Include a section on parameter identification, describing the methods used to determine the values of key parameters like suspension stiffness and damping coefficients. This could involve experimental data fitting or sensitivity analysis to ensure the model's accuracy.

3) Simulation Validation: The study relies heavily on simulations, but there is limited discussion on how these simulations were validated against real-world data. Suggestion: Conduct experimental tests with the actual XZJ5240JQZ30 vehicle or similar models to validate the simulation results. This would enhance the credibility of the findings and demonstrate the practical applicability of the proposed control system.

2. Technical aspects

1) Control System Design: The PID control system is used for optimization, but the choice of PID gains (P=8, I=5, D=1) seems arbitrary. Suggestion: Explain the process of tuning these gains, possibly using a systematic approach like Ziegler-Nichols method or model-based optimization techniques. This would provide a rationale for the chosen values and ensure optimal performance.

2) Handling of Nonlinearities: The control system may face challenges in handling the nonlinearities inherent in vehicle dynamics, such as tire force saturation or suspension nonlinearities. Suggestion: Explore the use of advanced control techniques like sliding mode control or model predictive control, which are better suited for nonlinear systems. These methods can provide robustness and improved performance under varying conditions.

3) Real-time Implementation: The study focuses on simulation results, but there is no discussion on the real-time implementation of the control system. Suggestion: Address the computational requirements and feasibility of implementing the control algorithm in real-time on the vehicle's onboard systems. Consider factors like processing power, response time, and sensor data availability.

4) Robustness to Disturbances: The impact of external disturbances, such as road irregularities or wind gusts, on the vehicle's stability is not explicitly considered. Suggestion: Incorporate disturbance rejection techniques in the control system design, such as using disturbance observers or robust control strategies, to ensure stability and performance in the presence of real-world uncertainties.

Below are my comments and suggestions to improve the writing and presentation of the manuscript.

1. Title and Abstract

Title: The title is quite lengthy. Consider simplifying it to highlight the core content and innovation of the study. For example, change "Study on optimization control of rescue hoist vehicle safety performance based on state feedback: A research on the XZJ5240JQZ30 All-Terrain Rescue Lift Vehicle" to "Optimization Control of All-Terrain Rescue Lift Vehicle Safety Performance Based on State Feedback".

Abstract: The abstract lacks a brief description of the research methods. It is suggested to add a sentence about how the 2DOF vehicle model and state-feedback PID optimization control system were established.

2. Introduction

Background Context: The introduction could benefit from a clearer linkage between the background information and the specific objectives of this study. Consider adding a paragraph that explicitly states how the existing challenges in rescue vehicle performance motivated this research.

Literature Review: While several studies are cited, there is room for a more comprehensive review. Include a

broader range of relevant literature to establish the gap your study aims to fill. For instance, discuss more extensively how previous research has addressed the stability and steering control of heavy vehicles in off-road conditions.
3. Methodology
Mathematical Models: The mathematical models and equations are central to the study. Ensure that all symbols and variables are clearly defined and consistently used throughout the text. Consider providing a table summarizing the key parameters and their units for easy reference.
Simulation Setup: The description of the simulation experiments could be more detailed. Specify the exact parameters and conditions used in the TruckSim and Simulink simulations, such as the exact specifications of the road surface and environmental conditions. This will enhance the reproducibility of the study.
4. Results
Data Presentation: While the results are presented, they could be more effectively visualized. Consider using additional graphs or charts to compare the performance metrics of the 4WS and FWS systems more clearly. For example, a side-by-side comparison of yaw rate and lateral offset for both systems at different speeds would be helpful.
Statistical Analysis: Include statistical analysis to quantify the significance of the differences observed between the 4WS and FWS systems. This will strengthen the validity of your findings.
5. Discussion
Interpretation of Results: The discussion section should more thoroughly interpret the results in the context of the research objectives. Explain how the findings contribute to the understanding of rescue vehicle stability and steering control, and how they can be applied in practical scenarios.
Limitations: Address the limitations of the study more explicitly. Discuss any assumptions made in the model or simulations that might affect the results, and suggest areas for future research to address these limitations.
6. Conclusion
Summary of Findings: The conclusion should succinctly summarize the key findings and their implications. Clearly state how the state-feedback PID optimization control system enhances the stability of the rescue lift vehicle.
Future Work: Suggest potential directions for future research. For example, consider how the control system could be adapted for different types of rescue vehicles or tested under more varied environmental conditions.
7. Writing and Language
Clarity and Conciseness: Some sections contain lengthy sentences that could be broken down for clarity. Aim for concise and straightforward language to improve readability.
Technical Jargon: Ensure that technical terms and jargon are explained or defined for readers who may not be experts in the field. This will make the paper more accessible to a broader audience.
8. Figures and Tables
Figure Quality: Ensure that all figures are of high quality and clearly labeled. The captions should provide sufficient information to understand the figures without referring to the text.
Table Organization: If tables are used, ensure they are well-organized and easy to read. Use consistent formatting and include clear headings and units.
By addressing these comments and suggestions, the manuscript can be strengthened and made more suitable for publication.

Author Responses to Initial Comments:

Reviewer # 1 (Remarks to the Author):

Reply: We sincerely appreciate your meticulous review of our manuscript and the constructive feedback you have

provided. We have provided detailed responses to the issues you raised and have made revisions to address the shortcomings in the manuscript. We look forward to your review of our revised submission and your valuable feedback.

1. The images in the text did not display properly. Please ensure that there are no formatting errors in the final submitted version.

Reply: We sincerely apologize for the inconvenience caused by our oversight, which affected your reading experience. During the revision process, we have adjusted all the images included in the manuscript. We hope these modifications will enhance your reading experience.

For this comment, the relevant changes are shown in the new version of the figure.

2. Table 1 should have 3 columns, each column consisting of parameter name, parameter meaning, and parameter value.

Reply: During the revision process, we have added the definitions of various parameters in Table 1. These modifications will help readers better understand each parameter.

For this comment, the revised manuscript is shown below, with the modified sections marked in blue.

Table 1 Basic Parameters of the All-Terrain Rescue Lift Vehicle

Parameter Name	Parameter meaning	Parameter Value
Vehicle mass	Total vehicle weight	24000kg
Vehicle length	Total length of the vehicle	11575mm
Vehicle width	Total width of the vehicle	2500mm
Vehicle height	Total height of the vehicle	3855mm
Wheel base	Distance between front and rear axles	3505mm
Distance from CG to front wheel axle	Horizontal distance from CG to front axle	1935mm
Center of mass height	Vertical height of the center of mass	1650mm
Tire specification	Tire model and load capacity	385/95R25 170F

3. In the "Steering control system optimization design", a Linear 2-degree-of-freedom model was adopted, which means that this article only considers sideslip and yaw at the theoretical level, without considering roll and pitch. But the rollover caused by vehicle roll is also a serious problem. Is the dynamic model used in this article reasonable?

Reply: Thank you for your highly constructive comments. The selection of the linear 2DOF model in this study was based on actual vehicle parameters and accident occurrence rates. Given that the all-terrain rescue lift vehicle has a relatively low center of mass and a large weight, sideslip and yaw are the more common accident phenomena, whereas reports of roll and pitch accidents are relatively rare. Additionally, data obtained from a mining group in China support this observation. Therefore, while we acknowledge that, as you mentioned, vehicle rollover due to tilting is also a serious issue, from the perspective of addressing practical engineering problems, it is reasonable to focus solely on the analysis and optimization of sideslip and yaw phenomena.

For this comment, no modifications were necessary in the relevant part of the manuscript.

4. It is recommended to conduct tests under classic serpentine and hook conditions to verify the effectiveness of the proposed method in this paper.

Reply: Thank you for your valuable comments. The simulation conditions in this study were designed based on the actual conditions of mining roads.Since the vehicles operating on mining roads are mostly large and heavy mining

engineering vehicles, the road design, based on their intended use and the actual conditions of the mining site, does not involve serpentine conditions with continuous turns. Instead, the planned turning routes are relatively smooth. However, even under such conditions, these large and heavy mining vehicles still experience sideslip and yaw during turning. This study was conducted based on such an engineering application scenario.

For this comment, no modifications were necessary in the relevant part of the manuscript.

5. For experimental results, in addition to visualized curves, it is recommended to provide quantifiable indicator results.

Reply: To improve the presentation of the experimental results, we have optimized the images to provide a clearer comparison of the results. Additionally, to facilitate quantitative analysis, we have created a table displaying the specific peak values of the curves under both 4WS and FWS conditions. This allows for a clearer demonstration of the effectiveness of the proposed PID control method in enhancing the stability of the all-terrain rescue lift vehicle.

For this comment, the revised manuscript is shown below, with the modified sections marked in blue.

4.3. Analysis of vehicle stability at high speeds

In emergency situations, the rescue team needs to drive the all-terrain rescue lift vehicle at high speeds while ensuring both safety and maneuverability. Therefore, in this simulation experiment, the vehicle speed was set to 70 km/h to compare and analyze the performance parameters of the four-wheel steering (4WS) all-terrain rescue lift vehicle with PID control and the FWS all-terrain rescue lift vehicle without PID control. The results are shown in Figure 12.

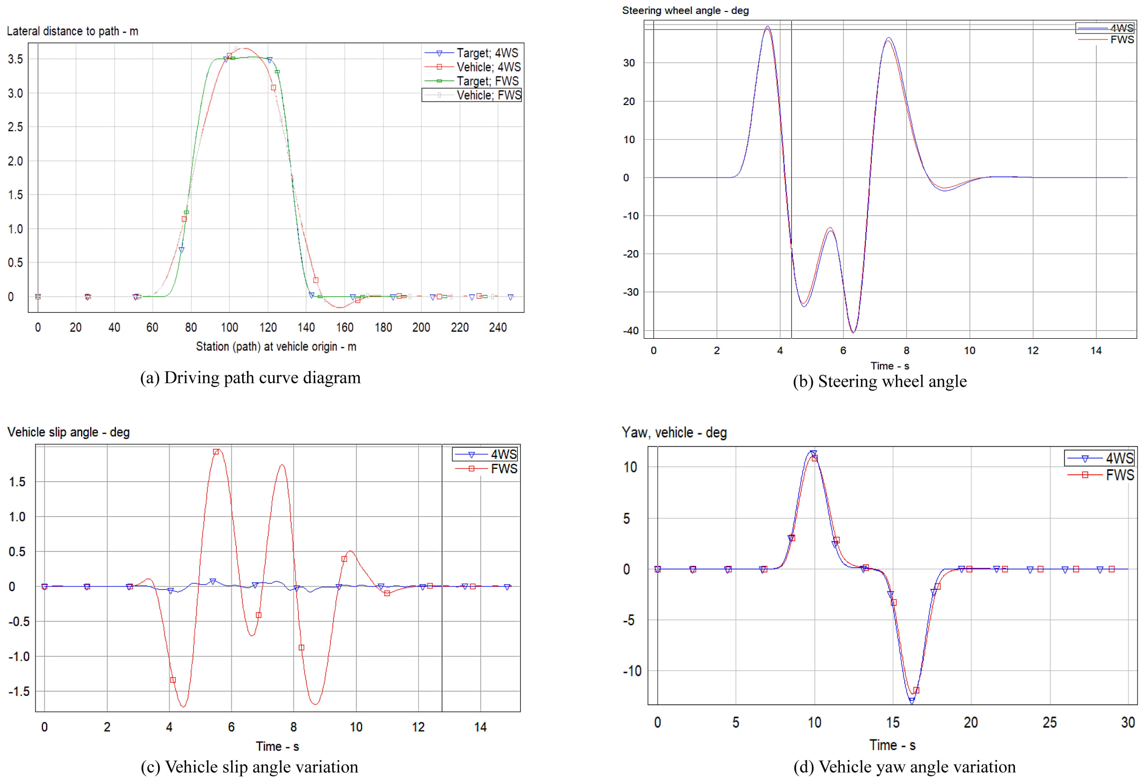

(a) Driving path curve diagram

(b) Steering wheel angle

(c) Vehicle slip angle variation

(d) Vehicle yaw angle variation

Fig. 12. Parameter curve changes at high speed

As illustrated in Figure 12(a), during high-speed driving, the trajectory of the 4WS vehicle with PID control exhibits a slight deviation compared to the FWS vehicle without PID control from the initial steering phase until 3.2 seconds. However, from 3.2 seconds until the conclusion of the simulation, the 4WS vehicle with PID control closely adheres to the intended path. In high-speed driving scenarios, PID control demonstrates a substantial stabilizing effect on the all-terrain rescue lift vehicle, particularly on curved road sections. To conduct a quantitative

analysis of the simulation results, the peak values of the steering wheel angle, vehicle slip angle, and vehicle yaw angle were extracted and are presented in Table 3.

Table 3 Peak data at high speed

Variable Name	4WS	FWS
Steering wheel angle (deg)	39, -41	38, -41
Vehicle slip angle (deg)	0.2, -0.2	1.9, -1.7
Vehicle yaw angle (deg/s)	12, -14	11, -13

A comprehensive analysis of Figures 12(b), 12(c), 12(d), and Table 3 demonstrates that under high-speed driving conditions, the 4WS vehicle effectively controls the vehicle slip angle, significantly reducing its positive and negative peak values compared to the FWS vehicle. Steering perception, being a subjective experience for the driver during vehicle turning, can be reflected in the vehicle's yaw angle response during cornering. In the given Double lane-change scenario, the yaw angle of the 4WS vehicle closely matches that of the FWS vehicle, ensuring consistent road feel for the driver, regardless of PID control application. However, at high speeds, the 4WS vehicle's steering wheel angle response does not show substantial improvements over the FWS vehicle, indicating that the effect of independent rear-wheel steering is not particularly pronounced under these conditions. This is primarily attributed to the vehicle's large mass, which results in a delayed response in independent rear-wheel steering control during high-speed driving.

4.4. Analysis of vehicle stability at low speeds

Under typical operational conditions, the all-terrain rescue lift vehicle primarily functions at reduced velocities. Consequently, this simulation experiment set the vehicle speed to 30 km/h to compare and analyze the performance parameters of the four-wheel steering (4WS) all-terrain rescue lift vehicle with PID control against the FWS all-terrain rescue lift vehicle without PID control. The comparative results are presented in Figure 13.

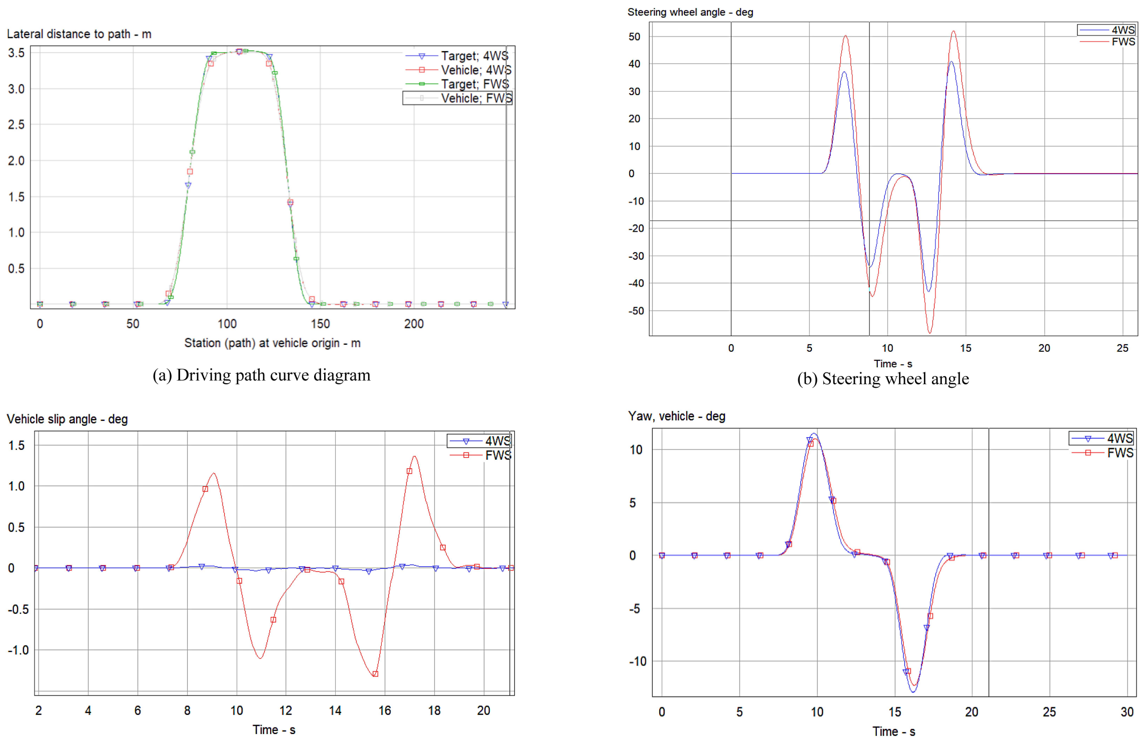

(a) Driving path curve diagram

(b) Steering wheel angle

(c) Vehicle slip angle variation

(d) Vehicle yaw angle variation

Fig. 13. Parameter curve changes at low speed

As illustrated in Figure 13(a), under low-speed driving conditions, the trajectory of the 4WS vehicle with PID control demonstrates superior alignment with the simulated path, exhibiting an enhanced tracking effect compared

to high-speed scenarios. To quantitatively assess the simulation outcomes, the maximum values of the steering wheel angle, vehicle slip angle, and vehicle yaw angle were extracted and are presented in Table 4.

Table 4 Peak data at low speed

Variable Name	4WS	FWS
Steering wheel angle (deg)	42, -44	53, -60
Vehicle slip angle (deg)	0.1, -0.1	1.4, -1.3
Vehicle yaw angle (deg/s)	12, -14	11, -13

A thorough examination of Figures 13(b), 13(c), 13(d), and Table 4 indicates that at lower velocities, the enhancement in vehicle slip angle is more pronounced, exhibiting notable decreases at multiple peak points. Consistent with previous findings, the yaw angle of both 4WS and FWS vehicles remains largely unchanged, ensuring a uniform road feel for the driver, irrespective of PID control application. Furthermore, the study observed a substantial reduction in the peak positive and negative values of the steering wheel angle for the 4WS vehicle with PID control. This observation suggests that during active steering, the 4WS all-terrain rescue lift vehicle can execute more efficient steering maneuvers without necessitating large steering wheel angles.

4.5. Analysis of the influence of center of mass height on vehicle stability

The all-terrain rescue lift vehicle examined in this study incorporates a chassis suspension with a maximum travel of 262 mm. While the long-travel suspension substantially improves the vehicle's off-road performance, it also impacts driving stability. Consequently, this simulation experiment was conducted at a velocity of 30 km/h utilizing the PID-controlled 4WS vehicle model. Under the Double lane-change scenario, two center-of-mass heights, 1650 mm and 1910 mm, were evaluated, with the results presented in Figure 14.

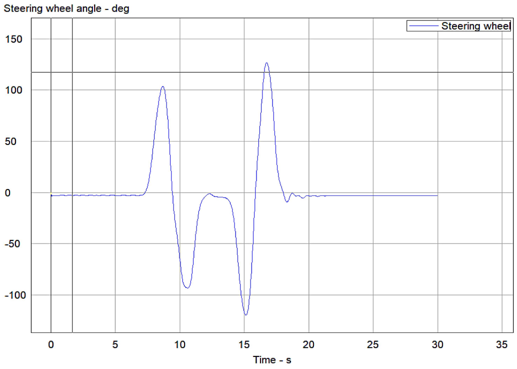

(a) Steering wheel angle of lower center-of-mass

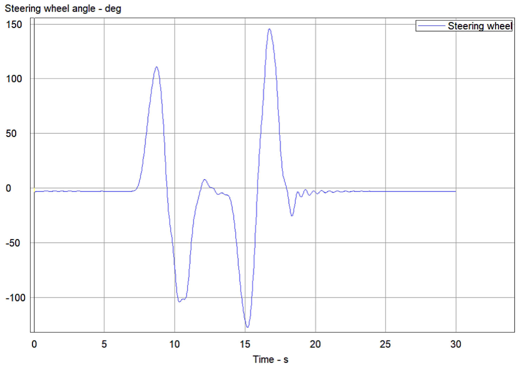

(b) Steering wheel angle of higher center-of-mass

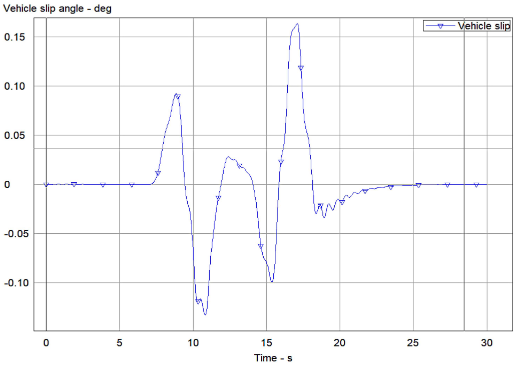

(c) Vehicle slip angle of lower center-of-mass

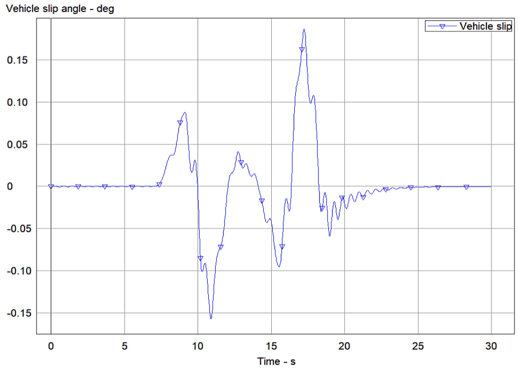

(d) Vehicle yaw angle of higher center-of-mass

Fig. 14. Parameter curve changes at different center-of-mass heights

For a quantitative analysis of the simulation results, the maximum values of the steering wheel angle and vehicle slip angle were documented, as presented in Table 5.

Table 5 Peak data at different center-of-mass heights

Variable Name	Higher center-of-mass	Lower center-of-mass

Steering wheel angle (deg)	145.5, -127.2	126.8, -119.8
Vehicle slip angle (deg)	0.18, -0.16	0.17, -0.13

A thorough examination of Figure 14 and Table 5 reveals notable distinctions in steering wheel angles between the two center-of-mass heights. The positive and negative peak values for the lower center-of-mass 4WS vehicle are considerably reduced compared to those of the higher center-of-mass vehicle. Furthermore, after 18 seconds of operation, the steering wheel angle of the higher center-of-mass vehicle demonstrates approximately 7 seconds of oscillation, with a maximum difference of 15 degrees. The overall variation trend of the vehicle slip angle remains consistent for both high and low center-of-mass vehicles. However, the slip angle of the lower center-of-mass vehicle exhibits greater stability and improved convergence relative to the higher center-of-mass vehicle. These observations suggest that a lower center of mass enhances vehicle steering stability and reduces the steering wheel angle required for effective maneuvering.

6. Why is PID used as the optimization controller in the article, and what are the advantages of this method compared to other methods?

Reply: The PID controller is a classic optimization control model that regulates based on the Proportional, Integral, and Derivative components of the deviation. It offers advantages such as low computational complexity and fast response speed, making it well-suited for large mining engineering vehicles like the all-terrain rescue lift vehicle. Additionally, since PID control is not model-based, it can be more easily applied to other large mining engineering vehicles similar to the all-terrain rescue lift vehicle, thereby reducing costs. These are the reasons why this study selects PID as the optimization controller.

For this comment, no modifications were necessary in the relevant part of the manuscript.

7. This article uses existing dynamic models and control methods, and the innovation of this article is not clear enough.

Reply: Indeed, as you have pointed out, the dynamic model and control method used in this study have been reported in previous research. However, our study focuses on addressing practical issues encountered in engineering applications. Specifically, we analyze the problems of sideslip and yaw during the operation of the XZJ5240JQZ30 all-terrain rescue lift vehicle and subsequently optimize the design of its chassis and suspension. Furthermore, based on the 2DOF vehicle model, we have developed a state-feedback PID optimization control system, which reduces the vehicle's centroid slip angle and yaw rate during steering, thereby enhancing its stability and passing ability.

For this comment, no modifications were necessary in the relevant part of the manuscript.

8. The Introduction section lacks a comprehensive summary of existing research. It is recommended to cite "A review on reinforcement learning based highway autonomous vehicle control" and "Longitudinal and lateral control methods from single vehicle to autonomous platform".

Reply: During the revision process, we have added citations and discussions for the articles "A Review on Reinforcement Learning-Based Highway Autonomous Vehicle Control" and "Longitudinal and Lateral Control Methods from Single Vehicle to Autonomous Platform." Additionally, to provide a more comprehensive summary of existing research, we have incorporated the following references.

[1] Irshayyid A, Chen J, Xiong G. A Review on Reinforcement Learning-based Highway Autonomous Vehicle Control[J]. Green Energy and Intelligent Transportation, 2024: 100156.

[2] Spielberg N A, Brown M, Kapania N R, et al. Neural network vehicle models for high-performance automated driving[J]. Science robotics, 2019, 4(28): eaaw1975.

[3] Zheng Z, Ye Z, Zheng X. Intelligent vehicle lateral control strategy research based on feedforward+ predictive LQR algorithm with GA optimisation and PID compensation[J]. Scientific Reports, 2024, 14(1): 22317.
[4] Ariff M H M, Zamzuri H, Nordin M A M, et al. Optimal control strategy for low speed and high speed four-wheel-active steering vehicle[J]. Journal of Mechanical Engineering and Sciences, 2015, 8: 1516-1528.
[5] Zhang Y, Wang Z, Wang Y, et al. Research on automobile four-wheel steering control system based on yaw angular velocity and centroid cornering angle[J]. Measurement and Control, 2022, 55(1-2): 49-61.
[6] Song L, Li J, Wei Z, et al. Longitudinal and lateral control methods from single vehicle to autonomous platoon[J]. Green Energy and Intelligent Transportation, 2023, 2(2): 100066.
[7] Mokbel E F F, M Abd-El-Tawwab A, Mourad M, et al. Improving Handling Performance of a Four-Wheel Steering Vehicles Using LQR Controller[J]. Journal of Advanced Engineering Trends, 2024, 43(2): 509-516.
[8] Xu J, Sui Z, Xu F, et al. A Novel Model-Free Adaptive Proportional–Integral–Derivative Control Method for Speed-Tracking Systems of Electric Balanced Forklifts[J]. Applied Sciences, 2023, 13(23): 12816.
[9] Xu J, Sui Z, Xu F, et al. A Novel Model-Free Adaptive Proportional–Integral–Derivative Control Method for Speed-Tracking Systems of Electric Balanced Forklifts[J]. Applied Sciences, 2023, 13(23): 12816.
[10] He Y, Xiao B. Research of the forklift power-assisted steering system based on safety steering speed control[J]. International Journal On Smart Sensing and Intelligent Systems, 2015, 8(1): 749-765.
[11] Jiang Z, Xiao B. Electric power steering system control strategy based on robust H∞ control for electric forklift[J]. Mathematical Problems in Engineering, 2018, 2018(1): 7614304.

For this comment, the revised manuscript is shown below, with the modified sections marked in blue.

During the Chilean mine rescue operation, scholars proposed a concept of utilizing drilling technology for rescue purposes [1-2]. They analyzed potential emergency scenarios and operational details, establishing a foundation for the development of large-diameter drilling technology. Qian Q et al. [3] introduced a novel rescue lift vehicle with a design that enhanced overall integrity and correlation, improving rescue efficiency. As technology advances, vehicle intelligence has progressively improved. Irshayyid A et al. [4] highlighted that Deep Reinforcement Learning (DRL) presents promising opportunities for training autonomous vehicles to manage complex driving tasks. Spielberg N A et al. [5] introduced a physics model-driven neural network structure for vehicle automation. This model can accurately predict road surface conditions without explicitly calculating road friction, even on varying terrains. To address the demands of modern mining rescue missions, which require higher intelligence levels and enhanced off-road capabilities for rescue equipment, it is essential to develop rescue lift devices with increased intelligence and superior adaptability to harsh road conditions [6-7]. Pilutti T et al. [8] pioneered the use of differential braking to analyze vehicle steering stability. Doumiati M et al. [9] proposed a control system that stabilizes vehicles to achieve desired yaw rates through collaborative steering and braking efforts, demonstrating the effectiveness of the linear matrix inequality (LMI) control scheme in critical driving conditions. Aouadj N et al. [10] enhanced vehicle maneuverability and stability by coordinating active front steering and direct yaw control, ensuring improved steering performance while maintaining stability in extreme driving scenarios. Zheng Z et al. [11] proposed a feedforward + predictive Linear Quadratic Regulator (LQR) lateral motion control algorithm based on Genetic Algorithm (GA) parameter optimization and PID steering angle compensation, enhancing vehicle tracking accuracy and stability. The simulation results of a case study demonstrated that LQR and Model Predictive Control (MPC) could prevent rollover with a relatively rapid response when the forklift carries a half load and moves at a slow speed. Ariff M H M et al. [12] proposed a Four-Wheel Active Steering (4WAS) system applicable to both low-speed and high-speed operations based on optimal control theory. The results showed that, at low speeds, the turning radius was reduced compared to Front-Wheel Steering (FWS) vehicles. Zhang Y et al. [13], based on quadratic optimal control theory, proposed an optimal control approach for a four-wheel steering system and conducted simulations using MATLAB/Simulink.

The results indicated that the optimal control-based 4WS system could enhance vehicle slip and yaw stability. A review of existing research reveals that current studies primarily utilize optimization control methods such as PID, LQR, and Robust H-infinity Control [14-16] to improve vehicle steering stability. However, these studies mainly focus on passenger cars and small construction vehicles [17-19], with limited research on large-scale mining engineering vehicles such as all-terrain rescue lift vehicles.

Reviewer # 2 (Remarks to the Author):

The manuscript investigates the steering stability of the XZJ5240JQZ30 all-terrain rescue lift vehicle under Double Line Change conditions. It develops a state-feedback PID optimization control system based on a two-degree-of-freedom vehicle model to enhance the vehicle's stability during steering maneuvers. Simulation results demonstrate that the proposed control system effectively improves the vehicle's stability and steering performance at both high and low speeds.

Reply: We sincerely appreciate your meticulous review of our manuscript and the constructive feedback you have provided. We have provided detailed responses to the issues you raised and have made revisions to address the shortcomings in the manuscript. We look forward to your review of our revised submission and your valuable feedback.

Below are comments and suggestions.

1. Methodological aspects

1) Model Assumptions: The linear two-degree-of-freedom (2DOF) model is used for vehicle stability analysis. While this model is a good starting point, it may not fully capture the complex dynamics of a heavy rescue vehicle, especially under extreme conditions. Suggestion: Consider incorporating a more sophisticated model, such as a nonlinear model or a 3DOF model, to better represent the vehicle's behavior under various steering and speed scenarios.

Reply: The selection of the linear 2DOF model in this study was based on actual vehicle parameters and accident occurrence rates. Given that the all-terrain rescue lift vehicle has a relatively low center of mass and a large weight, sideslip and yaw are more common accident phenomena, while reports of roll and pitch accidents are less frequent. Additionally, data obtained from a mining group in China support this observation. Therefore, while adopting a more complex model, as you suggested, could better reflect the vehicle's behavior under various conditions, it is still reasonable to focus on analyzing and optimizing sideslip and yaw phenomena based on the engineering practicalities of this study.

For this comment, no modifications were necessary in the relevant part of the manuscript.

1. Methodological aspects

2) Parameter Identification: The paper does not detail how the parameters for the mathematical models were identified or validated. Suggestion: Include a section on parameter identification, describing the methods used to determine the values of key parameters like suspension stiffness and damping coefficients. This could involve experimental data fitting or sensitivity analysis to ensure the model's accuracy.

Reply: The determination of model parameters is crucial for numerical simulations. In response to the issue you raised, we have added a paragraph in the manuscript describing the setup of the model parameters. Additionally, we have performed a numerical simulation to validate the model, ensuring its accuracy.

For this comment, the revised manuscript is shown below, with the modified sections marked in blue.

4.1. Simulation parameter settings

For the simulation process, a 2A Euro Cab-over type model vehicle, which closely resembles the overall

appearance of the all-terrain rescue hoisting vehicle, was selected for numerical simulation. Based on the actual data of the XZJ5240JQZ30 all-terrain rescue lift vehicle, various fundamental parameters of the model vehicle were adjusted in Trucksim. The XZJ5240JQZ30 all-terrain rescue lift vehicle is equipped with an MC07H.33-60 engine and a 12AS1215S0 automated manual transmission. According to its actual parameters, the vehicle's control air intake in Trucksim was set to ≥ 1250 kg/h, with a pressure difference of ≤ 45 kPa, a maximum input torque of 1200 Nm, and a maximum input speed of 2650 rpm. The remaining specific vehicle parameters are shown in Table 2.

Table 2 Vehicle Model Parameters

Simulation parameters	Parameters setting
Vehicle quality	24000 kg
Vehicle length	11575 mm
Vehicle width	2500 mm
Tyre height	635 mm
Steering damping	4.0 Nm·s/deg
Hys reference angle	0.5 deg
Spin inertia for each sid	10.0 kg·m^2

To more accurately reflect the motion state of the XZJ5240JQZ30 all-terrain rescue lift vehicle on mine rescue roads, the simulation conditions were set to a Double lane-change scenario based on actual mine rescue road conditions. The road surface material was defined as dry asphalt, and the road adhesion coefficient was set to μ=0.85.

4.2. Validation of simulation

To verify the compatibility between the model built in Simulink based on theoretical formulas and the built-in model in Trucksim, it was necessary to perform a simulation validation of the ideal 2DOF model before conducting the Trucksim and Simulink co-simulation experiments. This ensures the reliability of the established XZJ5240JQZ30 all-terrain rescue lift vehicle model. Under the classic Double lane-change scenario, a sinusoidal input was applied for simulation validation, and the variations in the centroid sideslip angle and yaw rate were monitored. The results, shown in Figure 11, indicate that the motion trajectory of the linear 2DOF model built in Simulink closely follows the driving trajectory of the Trucksim model, effectively verifying the feasibility of the vehicle model.

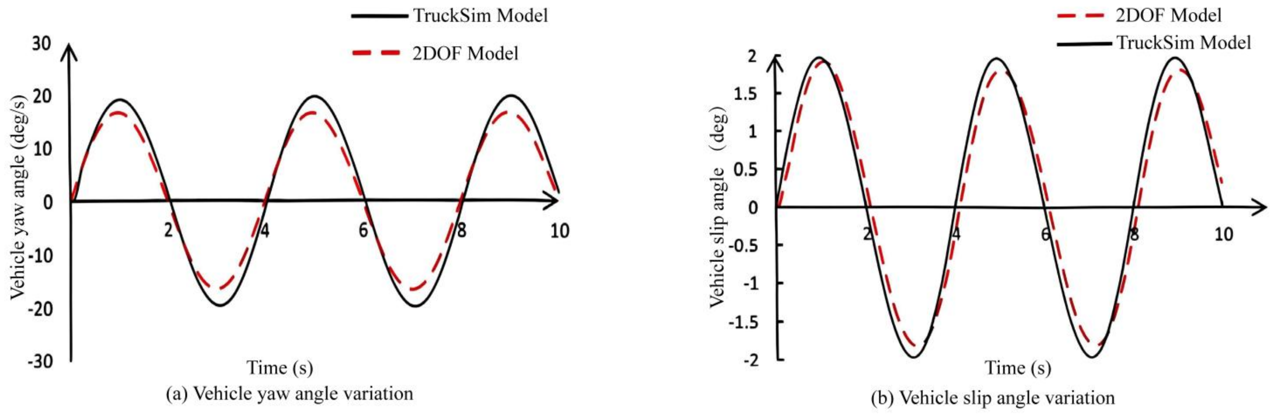

Fig. 11. Simulation Verification of the Vehicle Model

1. Methodological aspects

3) Simulation Validation: The study relies heavily on simulations, but there is limited discussion on how these

simulations were validated against real-world data. Suggestion: Conduct experimental tests with the actual XZJ5240JQZ30 vehicle or similar models to validate the simulation results. This would enhance the credibility of the findings and demonstrate the practical applicability of the proposed control system.

Reply: This study is based on engineering practice, and therefore, I believe your suggestion to conduct on-site experiments using the XZJ5240JQZ30 all-terrain rescue lift vehicle is necessary. As a result, we applied for and borrowed the site of a mining group in China to conduct real vehicle experiments. During the experiments, we measured the vehicle's steering system and tested the chassis and suspension structure. The results showed that the vehicle, after being improved with the PID optimization control model, meets the practical requirements for steering angles, with minimal steering errors and response delays. This effectively enhanced the stability of the all-terrain rescue lift vehicle. Additionally, the optimization of the chassis and suspension structure improved the road adaptability and terrain crossing ability of the XZJ5240JQZ30 all-terrain rescue lift vehicle.

For this comment, the revised manuscript is shown below, with the modified sections marked in blue.

5. Steering control experiment of the all-terrain rescue lift vehicle

Following the optimization of the vehicle structure and steering system, as well as the analysis of numerical simulation results, a steering control experiment is conducted to further evaluate the practical application value of the PID-optimized control system in enhancing vehicle stability. The experiment is carried out on emergency rescue roads in the auxiliary shaft area of the Meihua Mine using the XZJ5240JQZ30 all-terrain rescue lift vehicle.

The experiment recorded the theoretical steering angle, actual vehicle steering angle, and steering angle error, with the results depicted in Figure 15. The experimental findings demonstrate that the improved vehicle's actual steering angle generally satisfies practical requirements, exhibiting minimal steering error and response delay. The actual steering angle closely approximates the theoretical values, with the steering angle error reaching a negative peak of -3.5% at 20s and a maximum positive peak of 2.5% at 245s. These results corroborate the effectiveness of the PID-optimized control model proposed in this study in enhancing the stability of the all-terrain rescue lift vehicle.

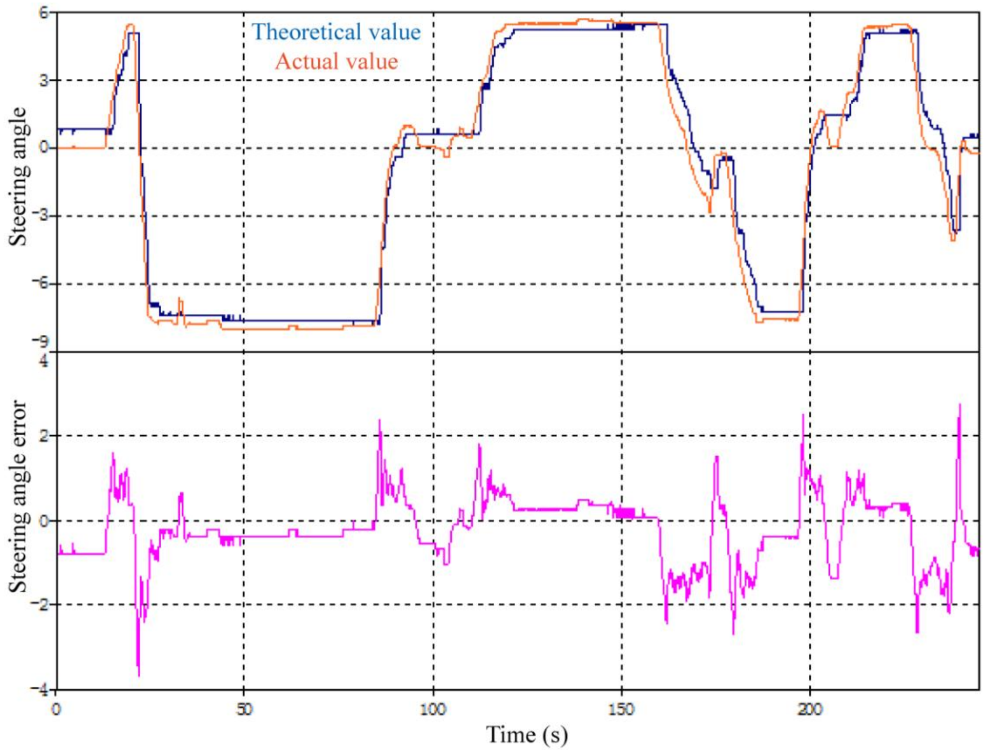

Fig. 15. Parameter curve changes in field tests

Concurrently, the optimized chassis and suspension structure proposed in this study underwent testing during the experiment. Through comparative analysis, the road adaptability and terrain traversal capability of the XZJ5240JQZ30 all-terrain rescue lift vehicle were assessed. Post-optimization, the vehicle's minimum turning diameter was reduced to 9.7 m, with the maximum outer wheel steering angle of the second axle reaching 24° and the maximum inner wheel steering angle reaching 32°.

2. Technical aspects

1) Control System Design: The PID control system is used for optimization, but the choice of PID gains (P=8, I=5, D=1) seems arbitrary. Suggestion: Explain the process of tuning these gains, possibly using a systematic approach like Ziegler-Nichols method or model-based optimization techniques. This would provide a rationale for the chosen values and ensure optimal performance.

Reply: The PID gain parameters in this study were not chosen arbitrarily. In the original manuscript, due to space limitations, we did not include a detailed description of the PID gain tuning process. In the revised version, we have added a detailed discussion of this process in the manuscript.

For this comment, the revised manuscript is shown below, with the modified sections marked in blue.

3.3. PID optimized control model

This control model employs the lateral offset of the center of gravity as the primary control variable. A PID control system adjusts the longitudinal forces of individual wheels to equilibrate the yaw moment required during steering. Figure 9 depicts the control model schematically.

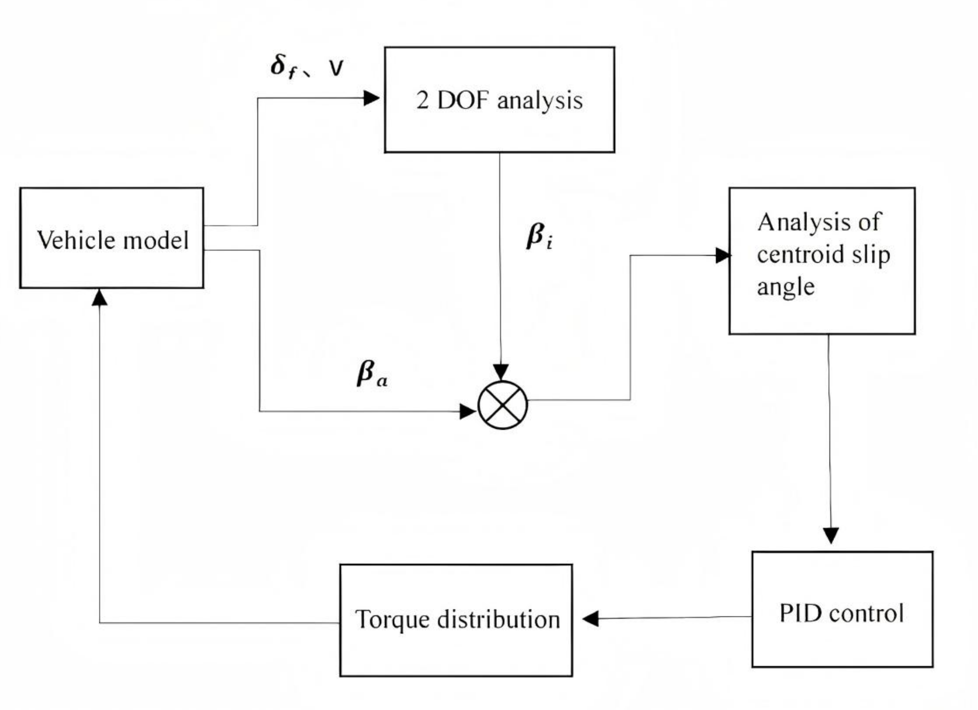

Fig. 9. Vehicle PID Control Model

The control model of the XZJ5240JQZ30 all-terrain rescue lift vehicle utilizes the steering wheel angle and speed as initial input conditions, establishing a linear 2DOF model to calculate the ideal vehicle slip angle while providing real-time feedback on the linear relationship between the vehicle slip angle and active lateral acceleration. The vehicle's operating state is determined by the deviation between the actual and ideal vehicle slip angle, thereby activating the PID control system. When the vehicle exhibits oversteering, the deviation in vehicle slip angle triggers the control system, which adjusts the vehicle's motion by generating the required additional yaw moment

and distributing it among the wheels. This torque distribution enhances the vehicle's stability during maneuvering.

To determine suitable PID gain parameters for the XZJ5240JQZ30 all-terrain rescue lift vehicle, the trial-and-error method was employed for parameter tuning. Initially, the integral and derivative constants were set to zero, and the proportional coefficient was gradually increased until oscillations appeared in the response curve. Adjustments continued until the oscillations disappeared, allowing the proportional constant to be determined. Following this approach, the integral and derivative constants were then tuned sequentially. Ultimately, the optimized PID parameters were obtained as P = 8, I = 5, and D = 1.

2. Technical aspects

2) Handling of Nonlinearities: The control system may face challenges in handling the nonlinearities inherent in vehicle dynamics, such as tire force saturation or suspension nonlinearities. Suggestion: Explore the use of advanced control techniques like sliding mode control or model predictive control, which are better suited for nonlinear systems. These methods can provide robustness and improved performance under varying conditions.

Reply: Thank you for your forward-looking suggestions. These recommendations have been very helpful for our research. However, due to the time constraints for revising the manuscript and the limited length of the paper, we may not be able to implement these suggestions in this version, and we hope you can understand. While this may introduce certain limitations to our study, we believe it will not affect the accuracy of the results presented. Moreover, in future research, we will conduct a more in-depth investigation into this issue to address its limitations as quickly as possible.

For this comment, no modifications were necessary in the relevant part of the manuscript.

2. Technical aspects

3) Real-time Implementation: The study focuses on simulation results, but there is no discussion on the real-time implementation of the control system. Suggestion: Address the computational requirements and feasibility of implementing the control algorithm in real-time on the vehicle's onboard systems. Consider factors like processing power, response time, and sensor data availability.

Reply: During the revision process of the manuscript, we included a brief real vehicle experiment to verify the practical applicability of the PID control system and vehicle structure optimization proposed in this study. However, due to the time required for site approval, as well as the need to coordinate multiple departments and relevant personnel, the scope of experiments that could be conducted within the revision period was limited. Additionally, some of the methods discussed in this paper are still at the theoretical research stage, with limited practical operational experience. Therefore, while the real vehicle experiment has preliminarily validated the feasibility of the methods proposed in this paper, there are still some issues that need to be addressed. This will be our primary research focus, and we hope that you will be able to see our research in this area in the future.

For this comment, no modifications were necessary in the relevant part of the manuscript.

2. Technical aspects

4) Robustness to Disturbances: The impact of external disturbances, such as road irregularities or wind gusts, on the vehicle's stability is not explicitly considered. Suggestion: Incorporate disturbance rejection techniques in the control system design, such as using disturbance observers or robust control strategies, to ensure stability and performance in the presence of real-world uncertainties.

Reply: Thank you for your constructive suggestions. As this research is still in its early stages, certain vehicle and environmental models have been simplified, resulting in a lack of analysis and discussion regarding the impact of external factors on vehicle stability. After careful consideration, we agree that this is indeed a valuable topic for

further investigation. Therefore, in our future research, we will thoroughly explore the impact of adverse external factors on the stability of the XZJ5240JQZ30 all-terrain rescue lift vehicle and seek potential solutions.

For this comment, no modifications were necessary in the relevant part of the manuscript.

Below are comments and suggestions to improve the writing and presentation of the manuscript.

1. Title and Abstract

Title: The title is quite lengthy. Consider simplifying it to highlight the core content and innovation of the study. For example, change "Study on optimization control of rescue hoist vehicle safety performance based on state feedback: A research on the XZJ5240JQZ30 All-Terrain Rescue Lift Vehicle" to "Optimization Control of All-Terrain Rescue Lift Vehicle Safety Performance Based on State Feedback".

Abstract: The abstract lacks a brief description of the research methods. It is suggested to add a sentence about how the 2DOF vehicle model and state-feedback PID optimization control system were established.

Reply: Thank you for your important suggestion. We have revised the title of the manuscript to make it more concise and to better highlight the core content and innovation of the research. Additionally, we have made appropriate changes to the abstract, incorporating details about the vehicle experiment and providing an explanation of the establishment of the 2DOF vehicle model and the state-feedback PID optimization control system.

For this comment, the revised manuscript is shown below, with the modified sections marked in blue.

Full title: Optimization control of all-terrain rescue lift vehicle safety performance based on state feedback

Short title: PID control and steering stability of the all-terrain rescue lift vehicle

Abstract: The all-terrain rescue lift vehicle is instrumental in mining emergency rescue operations, with its operational stability being of utmost importance. This study focuses on the XZJ5240JQZ30 all-terrain rescue lift vehicle, optimizing its vehicle structure and steering system. A linear 2DOF model and a PID gain model were developed based on actual vehicle parameters. A feedback system was employed to adjust the rear-wheel steering angle, enabling four-wheel steering (4WS) vehicle control. Numerical simulations were conducted using TruckSim and Simulink software. Utilizing the classic Double lane-change scenario as a test scenario, the study compared variations in the vehicle's centroid slip angle and yaw rate at different speeds, analyzing the impact of PID gain on steering stability. Moreover, the relationship between the centroid height and 4WS vehicle stability at low speeds was examined. Based on these findings, practical application tests were performed on the XZJ5240JQZ30 all-terrain rescue lift vehicle, obtaining relevant data on steering angle error. The results indicate that vehicles equipped with the PID-optimized control system demonstrate significantly higher steering stability than those without it. Furthermore, in practical applications, the actual steering angle closely aligns with the theoretical values. This demonstrates that the proposed optimized control system has substantial practical application value.

Key words: All-terrain rescue lift vehicle; Linear 2DOF model; PID control system; Double lane-change scenario; Steering stability; 4WS vehicle control

2. Introduction

Background Context: The introduction could benefit from a clearer linkage between the background information and the specific objectives of this study. Consider adding a paragraph that explicitly states how the existing challenges in rescue vehicle performance motivated this research.

Literature Review: While several studies are cited, there is room for a more comprehensive review. Include a broader range of relevant literature to establish the gap your study aims to fill. For instance, discuss more extensively how previous research has addressed the stability and steering control of heavy vehicles in off-road conditions.

Reply: To provide a clearer explanation of the motivation behind this research, we have outlined the issues of

sideslip and yaw in the XZJ5240JQZ30 all-terrain rescue lift vehicle during actual operation in the introduction. To address these issues and improve vehicle performance, we initiated this study. Additionally, to offer a more comprehensive summary of existing research, we have included the following references in the introduction.

[1] Irshayyid A, Chen J, Xiong G. A Review on Reinforcement Learning-based Highway Autonomous Vehicle Control[J]. Green Energy and Intelligent Transportation, 2024: 100156.

[2] Spielberg N A, Brown M, Kapania N R, et al. Neural network vehicle models for high-performance automated driving[J]. Science robotics, 2019, 4(28): eaaw1975.

[3] Zheng Z, Ye Z, Zheng X. Intelligent vehicle lateral control strategy research based on feedforward+ predictive LQR algorithm with GA optimisation and PID compensation[J]. Scientific Reports, 2024, 14(1): 22317.

[4] Ariff M H M, Zamzuri H, Nordin M A M, et al. Optimal control strategy for low speed and high speed four-wheel-active steering vehicle[J]. Journal of Mechanical Engineering and Sciences, 2015, 8: 1516-1528.

[5] Zhang Y, Wang Z, Wang Y, et al. Research on automobile four-wheel steering control system based on yaw angular velocity and centroid cornering angle[J]. Measurement and Control, 2022, 55(1-2): 49-61.

[6] Song L, Li J, Wei Z, et al. Longitudinal and lateral control methods from single vehicle to autonomous platoon[J]. Green Energy and Intelligent Transportation, 2023, 2(2): 100066.

[7] Mokbel E F F, M Abd-El-Tawwab A, Mourad M, et al. Improving Handling Performance of a Four-Wheel Steering Vehicles Using LQR Controller[J]. Journal of Advanced Engineering Trends, 2024, 43(2): 509-516.

[8] Xu J, Sui Z, Xu F, et al. A Novel Model-Free Adaptive Proportional–Integral–Derivative Control Method for Speed-Tracking Systems of Electric Balanced Forklifts[J]. Applied Sciences, 2023, 13(23): 12816.

[9] Xu J, Sui Z, Xu F, et al. A Novel Model-Free Adaptive Proportional–Integral–Derivative Control Method for Speed-Tracking Systems of Electric Balanced Forklifts[J]. Applied Sciences, 2023, 13(23): 12816.

[10] He Y, Xiao B. Research of the forklift power-assisted steering system based on safety steering speed control[J]. International Journal On Smart Sensing and Intelligent Systems, 2015, 8(1): 749-765.

[11] Jiang Z, Xiao B. Electric power steering system control strategy based on robust H∞ control for electric forklift[J]. Mathematical Problems in Engineering, 2018, 2018(1): 7614304.

For this comment, the revised manuscript is shown below, with the modified sections marked in blue.

During the Chilean mine rescue operation, scholars proposed a concept of utilizing drilling technology for rescue purposes [1-2]. They analyzed potential emergency scenarios and operational details, establishing a foundation for the development of large-diameter drilling technology. Qian Q et al. [3] introduced a novel rescue lift vehicle with a design that enhanced overall integrity and correlation, improving rescue efficiency. As technology advances, vehicle intelligence has progressively improved. Irshayyid A et al. [4] highlighted that Deep Reinforcement Learning (DRL) presents promising opportunities for training autonomous vehicles to manage complex driving tasks. Spielberg N A et al. [5] introduced a physics model-driven neural network structure for vehicle automation. This model can accurately predict road surface conditions without explicitly calculating road friction, even on varying terrains. To address the demands of modern mining rescue missions, which require higher intelligence levels and enhanced off-road capabilities for rescue equipment, it is essential to develop rescue lift devices with increased intelligence and superior adaptability to harsh road conditions [6-7]. Pilutti T et al. [8] pioneered the use of differential braking to analyze vehicle steering stability. Doumiati M et al. [9] proposed a control system that stabilizes vehicles to achieve desired yaw rates through collaborative steering and braking efforts, demonstrating the effectiveness of the linear matrix inequality (LMI) control scheme in critical driving conditions. Aouadj N et al. [10] enhanced vehicle maneuverability and stability by coordinating active front steering and direct yaw control, ensuring improved steering performance while maintaining stability in extreme driving scenarios. Zheng Z et al. [11] proposed a feedforward + predictive Linear Quadratic Regulator (LQR) lateral motion control algorithm based on Genetic Algorithm (GA) parameter optimization and PID steering angle

compensation, enhancing vehicle tracking accuracy and stability. The simulation results of a case study demonstrated that LQR and Model Predictive Control (MPC) could prevent rollover with a relatively rapid response when the forklift carries a half load and moves at a slow speed. Ariff M H M et al. [12] proposed a Four-Wheel Active Steering (4WAS) system applicable to both low-speed and high-speed operations based on optimal control theory. The results showed that, at low speeds, the turning radius was reduced compared to Front-Wheel Steering (FWS) vehicles. Zhang Y et al. [13], based on quadratic optimal control theory, proposed an optimal control approach for a four-wheel steering system and conducted simulations using MATLAB/Simulink. The results indicated that the optimal control-based 4WS system could enhance vehicle slip and yaw stability. A review of existing research reveals that current studies primarily utilize optimization control methods such as PID, LQR, and Robust H-infinity Control [14-16] to improve vehicle steering stability. However, these studies mainly focus on passenger cars and small construction vehicles [17-19], with limited research on large-scale mining engineering vehicles such as all-terrain rescue lift vehicles.

Mining areas encompass extensive territories with complex and variable road conditions within the pits. During rescue operations, vehicle steering stability is paramount. While advancements in mine road planning have generally eliminated the need for continuous or excessively sharp turns in rescue routes, the substantial mass of all-terrain rescue lift vehicles still poses a significant risk of sideslip and yaw accidents during turning maneuvers. This study aims to enhance the steering stability and road adaptability of these vehicles by investigating the factors affecting the steering stability of large-scale mining engineering vehicles and addressing existing issues. The XZJ5240JQZ30 all-terrain rescue lift vehicle was selected as the research subject, with optimizations made to both its structure and steering system. A linear 2DOF model and a PID gain model were developed for the vehicle. Numerical simulations were conducted using Trucksim and Simulink, employing a classic Double lane-change scenario to analyze the impact of PID gain on vehicle steering stability under varying speeds and centroid heights. Based on these findings, a practical application test was performed on the XZJ5240JQZ30 all-terrain rescue lift vehicle to obtain relevant steering angle error data. This research provides a theoretical foundation for the design and optimization of steering stability in all-terrain rescue lift vehicles.

3. Methodology

Mathematical Models: The mathematical models and equations are central to the study. Ensure that all symbols and variables are clearly defined and consistently used throughout the text. Consider providing a table summarizing the key parameters and their units for easy reference.

Simulation Setup: The description of the simulation experiments could be more detailed. Specify the exact parameters and conditions used in the TruckSim and Simulink simulations, such as the exact specifications of the road surface and environmental conditions. This will enhance the reproducibility of the study.

Reply: We have thoroughly checked the symbols and variables used in the mathematical models and equations to ensure consistency throughout the manuscript. Additionally, we have summarized them in a variable notation table for easy reference. Regarding the specific parameters used in the numerical simulations, we have added a paragraph in the manuscript during the revision process to describe the relevant parameters, aiming to enhance the reproducibility of this study.

For this comment, the revised manuscript is shown below, with the modified sections marked in blue.

Nomenclature

M^1	The distance between the intersection points of the two kingpin centerlines and the ground [mm]

I_Z	Moment of inertia [kg/m^3]
ω_r	Yaw rate [deg/s]
k_f	Front wheel stiffness [N/m]
k_r	Rear wheel stiffness [N/m]
β	Center of mass slip angle [deg]
δ	Front wheel steering angle [deg]
μ	Vehicle velocity in the x-axis direction [km/h]
υ	Vehicle velocity in the y-axis direction [km/h]

4.1. Simulation parameter settings

For the simulation process, a 2A Euro Cab-over type model vehicle, which closely resembles the overall appearance of the all-terrain rescue hoisting vehicle, was selected for numerical simulation. Based on the actual data of the XZJ5240JQZ30 all-terrain rescue lift vehicle, various fundamental parameters of the model vehicle were adjusted in Trucksim. The XZJ5240JQZ30 all-terrain rescue lift vehicle is equipped with an MC07H.33-60 engine and a 12AS1215S0 automated manual transmission. According to its actual parameters, the vehicle's control air intake in Trucksim was set to ≥ 1250 kg/h, with a pressure difference of ≤ 45 kPa, a maximum input torque of 1200 Nm, and a maximum input speed of 2650 rpm. The remaining specific vehicle parameters are shown in Table 2.

Table 2 Vehicle Model Parameters

Simulation parameters	Parameters setting
Vehicle quality	24000 kg
Vehicle length	11575 mm
Vehicle width	2500 mm
Tyre height	635 mm
Steering damping	4.0 Nm·s/deg
Hys reference angle	0.5 deg
Spin inertia for each sid	10.0 kg·m^2

To more accurately reflect the motion state of the XZJ5240JQZ30 all-terrain rescue lift vehicle on mine rescue roads, the simulation conditions were set to a Double lane-change scenario based on actual mine rescue road conditions. The road surface material was defined as dry asphalt, and the road adhesion coefficient was set to μ=0.85.

4. Results

Data Presentation: While the results are presented, they could be more effectively visualized. Consider using additional graphs or charts to compare the performance metrics of the 4WS and FWS systems more clearly. For example, a side-by-side comparison of yaw rate and lateral offset for both systems at different speeds would be helpful.

Statistical Analysis: Include statistical analysis to quantify the significance of the differences observed between the 4WS and FWS systems. This will strengthen the validity of your findings.

Reply: For the experimental results, we have optimized the presentation of the images to allow for a clearer comparison of the results. Additionally, to facilitate quantitative analysis, we have created a table. This table displays the specific peak values of the curves under both 4WS and FWS conditions, providing a clearer demonstration of the effect of the PID control method designed in this study on enhancing the stability of the all-terrain rescue lift vehicle.

For this comment, the revised manuscript is shown below, with the modified sections marked in blue.

4.3. Analysis of vehicle stability at high speeds

In emergency situations, the rescue team needs to drive the all-terrain rescue lift vehicle at high speeds while ensuring both safety and maneuverability. Therefore, in this simulation experiment, the vehicle speed was set to 70 km/h to compare and analyze the performance parameters of the four-wheel steering (4WS) all-terrain rescue lift vehicle with PID control and the FWS all-terrain rescue lift vehicle without PID control. The results are shown in Figure 12.

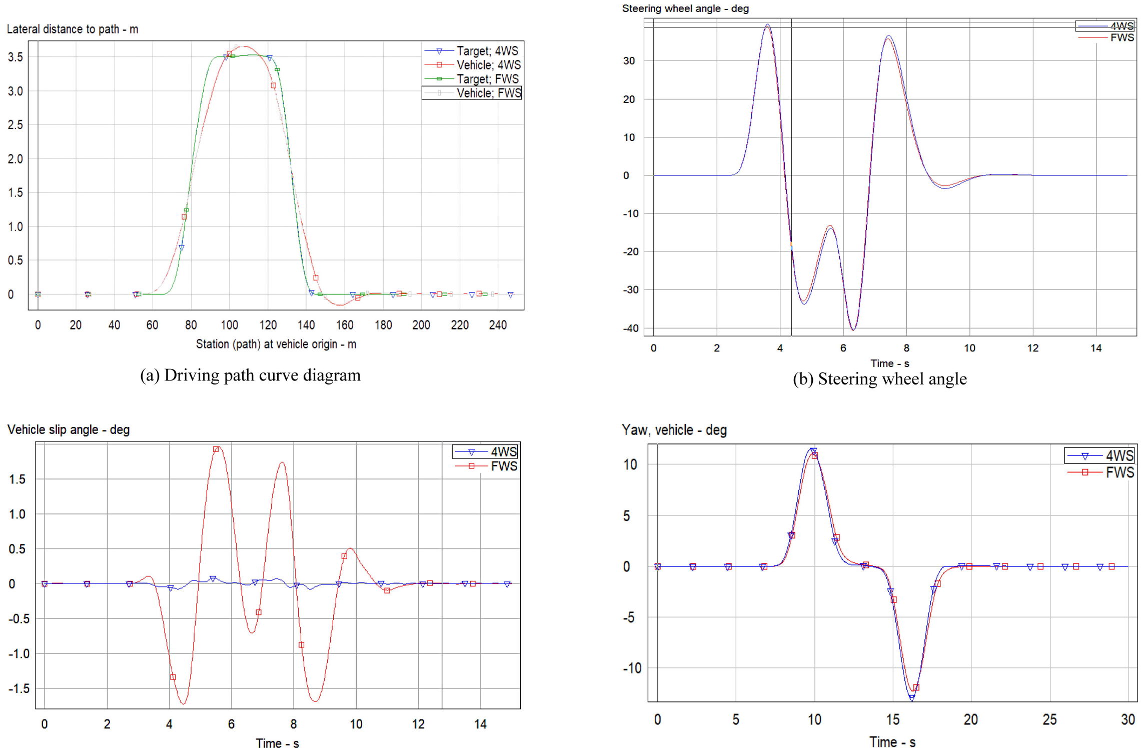

(a) Driving path curve diagram (b) Steering wheel angle

(c) Vehicle slip angle variation (d) Vehicle yaw angle variation

Fig. 12. Parameter curve changes at high speed

As illustrated in Figure 12(a), during high-speed driving, the trajectory of the 4WS vehicle with PID control exhibits a slight deviation compared to the FWS vehicle without PID control from the initial steering phase until 3.2 seconds. However, from 3.2 seconds until the conclusion of the simulation, the 4WS vehicle with PID control closely adheres to the intended path. In high-speed driving scenarios, PID control demonstrates a substantial stabilizing effect on the all-terrain rescue lift vehicle, particularly on curved road sections. To conduct a quantitative analysis of the simulation results, the peak values of the steering wheel angle, vehicle slip angle, and vehicle yaw angle were extracted and are presented in Table 3.

Table 3 Peak data at high speed

Variable Name	4WS	FWS
Steering wheel angle (deg)	39, -41	38, -41

Vehicle slip angle (deg)	0.2, -0.2	1.9, -1.7
Vehicle yaw angle (deg/s)	12, -14	11, -13

A comprehensive analysis of Figures 12(b), 12(c), 12(d), and Table 3 demonstrates that under high-speed driving conditions, the 4WS vehicle effectively controls the vehicle slip angle, significantly reducing its positive and negative peak values compared to the FWS vehicle. Steering perception, being a subjective experience for the driver during vehicle turning, can be reflected in the vehicle's yaw angle response during cornering. In the given Double lane-change scenario, the yaw angle of the 4WS vehicle closely matches that of the FWS vehicle, ensuring consistent road feel for the driver, regardless of PID control application. However, at high speeds, the 4WS vehicle's steering wheel angle response does not show substantial improvements over the FWS vehicle, indicating that the effect of independent rear-wheel steering is not particularly pronounced under these conditions. This is primarily attributed to the vehicle's large mass, which results in a delayed response in independent rear-wheel steering control during high-speed driving.

4.4. Analysis of vehicle stability at low speeds

Under typical operational conditions, the all-terrain rescue lift vehicle primarily functions at reduced velocities. Consequently, this simulation experiment set the vehicle speed to 30 km/h to compare and analyze the performance parameters of the four-wheel steering (4WS) all-terrain rescue lift vehicle with PID control against the FWS all-terrain rescue lift vehicle without PID control. The comparative results are presented in Figure 13.

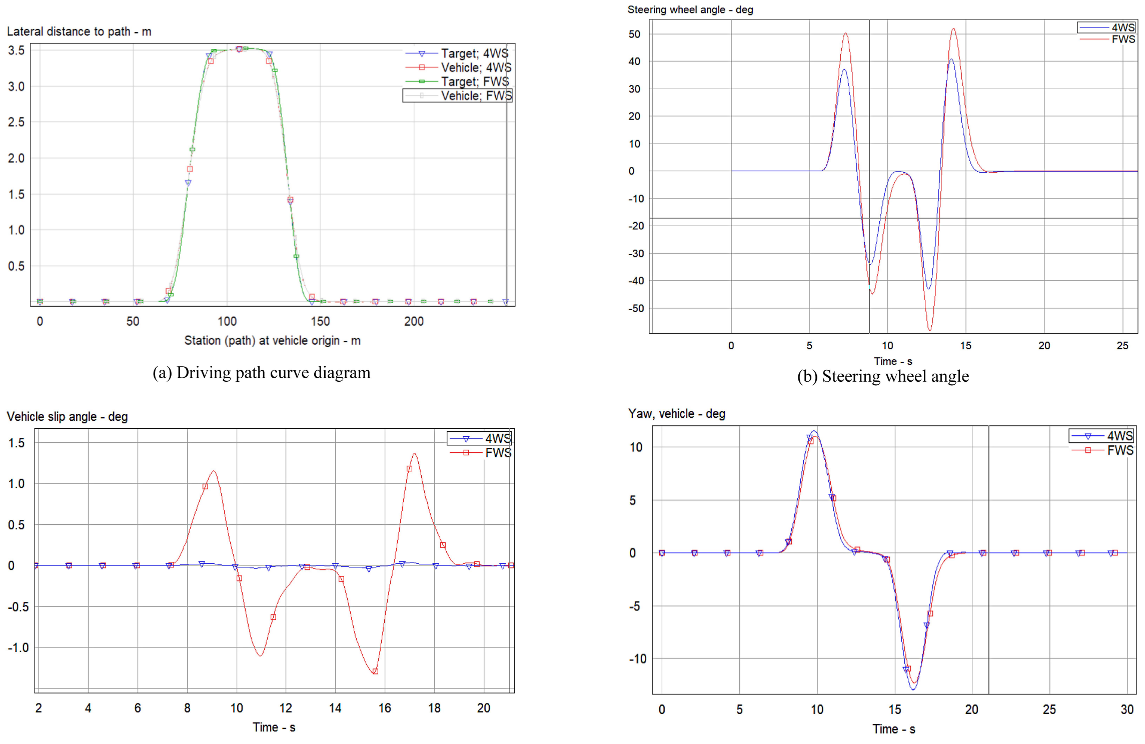

(a) Driving path curve diagram

(b) Steering wheel angle

(c) Vehicle slip angle variation

(d) Vehicle yaw angle variation

Fig. 13. Parameter curve changes at low speed

As illustrated in Figure 13(a), under low-speed driving conditions, the trajectory of the 4WS vehicle with PID control demonstrates superior alignment with the simulated path, exhibiting an enhanced tracking effect compared to high-speed scenarios. To quantitatively assess the simulation outcomes, the maximum values of the steering wheel angle, vehicle slip angle, and vehicle yaw angle were extracted and are presented in Table 4.

Table 4 Peak data at low speed

Variable Name	4WS	FWS
Steering wheel angle (deg)	42, -44	53, -60

Vehicle slip angle (deg)	0.1, -0.1	1.4, -1.3
Vehicle yaw angle (deg/s)	12, -14	11, -13

A thorough examination of Figures 13(b), 13(c), 13(d), and Table 4 indicates that at lower velocities, the enhancement in vehicle slip angle is more pronounced, exhibiting notable decreases at multiple peak points. Consistent with previous findings, the yaw angle of both 4WS and FWS vehicles remains largely unchanged, ensuring a uniform road feel for the driver, irrespective of PID control application. Furthermore, the study observed a substantial reduction in the peak positive and negative values of the steering wheel angle for the 4WS vehicle with PID control. This observation suggests that during active steering, the 4WS all-terrain rescue lift vehicle can execute more efficient steering maneuvers without necessitating large steering wheel angles.

4.5. Analysis of the influence of center of mass height on vehicle stability

The all-terrain rescue lift vehicle examined in this study incorporates a chassis suspension with a maximum travel of 262 mm. While the long-travel suspension substantially improves the vehicle's off-road performance, it also impacts driving stability. Consequently, this simulation experiment was conducted at a velocity of 30 km/h utilizing the PID-controlled 4WS vehicle model. Under the Double lane-change scenario, two center-of-mass heights, 1650 mm and 1910 mm, were evaluated, with the results presented in Figure 14.

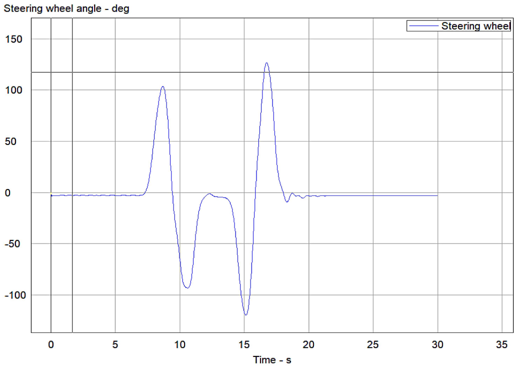

(a) Steering wheel angle of lower center-of-mass

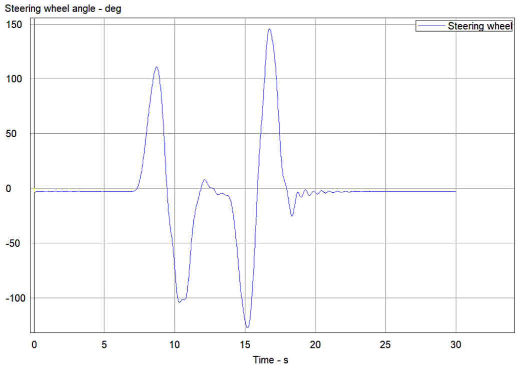

(b) Steering wheel angle of higher center-of-mass

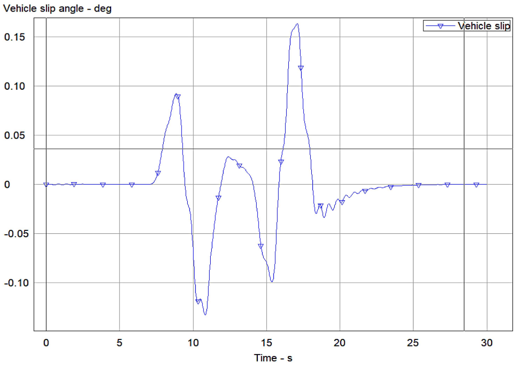

(c) Vehicle slip angle of lower center-of-mass

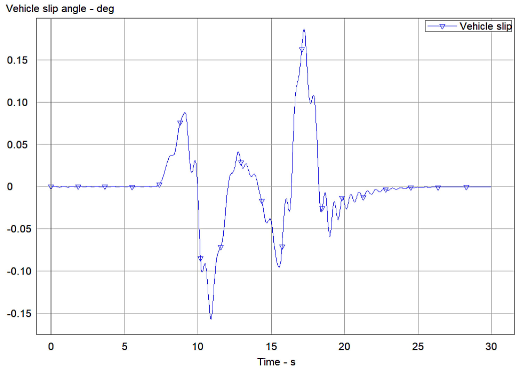

(d) Vehicle yaw angle of higher center-of-mass

Fig. 14. Parameter curve changes at different center-of-mass heights

For a quantitative analysis of the simulation results, the maximum values of the steering wheel angle and vehicle slip angle were documented, as presented in Table 5.

Table 5 Peak data at different center-of-mass heights

Variable Name	Higher center-of-mass	Lower center-of-mass
Steering wheel angle (deg)	145.5, -127.2	126.8, -119.8
Vehicle slip angle (deg)	0.18, -0.16	0.17, -0.13

A thorough examination of Figure 14 and Table 5 reveals notable distinctions in steering wheel angles between the two center-of-mass heights. The positive and negative peak values for the lower center-of-mass 4WS vehicle are considerably reduced compared to those of the higher center-of-mass vehicle. Furthermore, after 18 seconds of

operation, the steering wheel angle of the higher center-of-mass vehicle demonstrates approximately 7 seconds of oscillation, with a maximum difference of 15 degrees. The overall variation trend of the vehicle slip angle remains consistent for both high and low center-of-mass vehicles. However, the slip angle of the lower center-of-mass vehicle exhibits greater stability and improved convergence relative to the higher center-of-mass vehicle. These observations suggest that a lower center of mass enhances vehicle steering stability and reduces the steering wheel angle required for effective maneuvering.

5. Discussion

Interpretation of Results: The discussion section should more thoroughly interpret the results in the context of the research objectives. Explain how the findings contribute to the understanding of rescue vehicle stability and steering control, and how they can be applied in practical scenarios.

Limitations: Address the limitations of the study more explicitly. Discuss any assumptions made in the model or simulations that might affect the results, and suggest areas for future research to address these limitations.

Reply: In response to your suggestions, we have restructured the "Results and Discussion" section. We have provided a detailed explanation of the methods proposed in this study and their role in enhancing the steering stability and off-road capability of the vehicle, along with a description of the actual application scenarios. Additionally, based on the points mentioned in the previous feedback, "2. Technical aspects: (2) Handling of Nonlinearities (3) Real-time Implementation," we have discussed the limitations of the study.

For this comment, the revised manuscript is shown below, with the modified sections marked in blue.

6. Results and Discussion

This study optimizes the vehicle structure and steering system of the XZJ5240JQZ30 all-terrain rescue lift vehicle. A PID-optimized 4WS control system is implemented, which adjusts the longitudinal forces of individual wheels to balance the required yaw moment during steering, thereby enhancing both steering stability and off-road capability. Numerical simulations were conducted using TruckSim and Simulink to analyze the vehicle's performance under various conditions in the Double lane-change scenario. Additionally, field experiments are carried out to test and validate the feasibility of the proposed control system in real-world rescue operations.

The findings demonstrate that the PID-optimized control system substantially enhances vehicle stability. In high-speed scenarios, the PID-controlled 4WS system effectively minimizes the vehicle slip angle. In comparison to the conventional FWS system, the proposed control model maintains a more consistent vehicle trajectory, ensuring improved maneuverability and safety during rescue operations. In low-speed conditions, the 4WS system not only suppresses slip angle fluctuations but also decreases the required steering wheel angle, thus enhancing vehicle control in narrow and complex terrains. Additionally, the study examined the influence of center-of-mass height on stability, revealing that a lower center of mass improves steering stability and reduces steering wheel angle variations during turns. Based on these observations, steering control experiments were conducted on the all-terrain rescue lift vehicle, further corroborating the efficacy of the proposed PID control model. Experimental data revealed that the actual steering angles closely approximated theoretical values, with minimal error and response delay. This validates the viability of the PID-optimized 4WS system in practical applications, offering both theoretical and empirical references for enhancing all-terrain rescue lift vehicles.

Despite these promising results, the study presents certain limitations. The research on the control system remains insufficient, as it does not explicitly demonstrate the system's approach to addressing nonlinear dynamics in vehicle motion. Furthermore, the real-time implementation of the control algorithm on an onboard system has not been thoroughly investigated. To address these constraints, future research will concentrate on advanced control techniques for nonlinear systems and the real-time implementation of control algorithms on onboard systems. This focus aims to ensure practical applicability in emergency rescue scenarios.

6. Conclusion

Summary of Findings: The conclusion should succinctly summarize the key findings and their implications. Clearly state how the state-feedback PID optimization control system enhances the stability of the rescue lift vehicle.

Future Work: Suggest potential directions for future research. For example, consider how the control system could be adapted for different types of rescue vehicles or tested under more varied environmental conditions.

Reply: In response to this suggestion, we have added a "Main Conclusions" section in the manuscript. In this section, we summarize the key findings of the study and clearly outline the positive impact of the proposed method on enhancing the vehicle's steering stability and off-road capability. Furthermore, based on the points raised in the previous feedback, "2. Technical aspects: (4) Robustness to Disturbances" and "6. Conclusion: Future Work," we have discussed potential directions for future research.

For this comment, the revised manuscript is shown below, with the modified sections marked in blue.

7. Main Conclusions

Through the optimization of the chassis and suspension structure of the XZJ5240JQZ30 all-terrain rescue lift vehicle, a long-travel hydro-pneumatic suspension system was developed. This enhancement enables the vehicle to adapt more effectively to the challenging road conditions in mining areas, thereby substantially improving its off-road performance.

The PID-optimized four-wheel steering (4WS) control system proposed in this study significantly enhances the steering stability of the XZJ5240JQZ30 all-terrain rescue lift vehicle. This improvement is demonstrated through a reduction in vehicle slip angle and enhanced trajectory tracking capabilities under both high-speed and low-speed conditions. Furthermore, the research indicates that a lower center of mass contributes to improved vehicle maneuverability and stability. These findings offer valuable insights for future optimization of vehicle structure and steering control systems in similar applications.

Real-world application tests demonstrated that the PID-optimized 4WS control system exhibits excellent performance in practical scenarios. The observed steering angles closely aligned with the theoretical values, showing minimal error and response delay. These results validate the system's feasibility for implementation in actual rescue operations.

Subsequent research endeavors will concentrate on advancing control methodologies to more effectively manage the nonlinear dynamics inherent in rescue vehicles. Moreover, investigations will be undertaken to explore the real-time implementation of the control system on embedded in-vehicle platforms. Further evaluations under diverse environmental conditions will be conducted to validate the efficacy of the proposed system in authentic rescue scenarios.

7. Writing and Language

Clarity and Conciseness: Some sections contain lengthy sentences that could be broken down for clarity. Aim for concise and straightforward language to improve readability.

Technical Jargon: Ensure that technical terms and jargon are explained or defined for readers who may not be experts in the field. This will make the paper more accessible to a broader audience.

Reply: We have made certain revisions to address the language and expression issues in the manuscript. Additionally, regarding the technical terms used in the paper, we have added explanations and definitions where possible, without affecting the original structure of the discussion.

For this comment, the relevant changes are shown in the new version of the manuscript.

8. Figures and Tables

Figure Quality: Ensure that all figures are of high quality and clearly labeled. The captions should provide sufficient information to understand the figures without referring to the text.

Table Organization: If tables are used, ensure they are well-organized and easy to read. Use consistent formatting and include clear headings and units.

Reply: During the revision process, we carefully checked all the illustrations and tables included in the manuscript and made modifications to some of the tables and figures. These changes have helped improve the quality of the images and tables.

For this comment, the relevant changes are shown in the new version of the figure and table.

Thank you again for your time and consideration. Look forward to hearing from you.

Sincerely,

Dongmei Tian

Corresponding author: Dr. Dongmei Tian

E-mail address: tdm1999@126.com

Phone: +86 13463625956

Journal title: PLOS ONE

Article title: Optimization control of all-terrain rescue lift vehicle safety performance based on state feedback

Manuscript Number: PONE-D-24-59867
